# Hypoxia Inducible Factor-2α Regulates the Development of Retinal Astrocytic Network by Maintaining Adequate Supply of Astrocyte Progenitors

**DOI:** 10.1371/journal.pone.0084736

**Published:** 2014-01-27

**Authors:** Li-Juan Duan, Kotaro Takeda, Guo-Hua Fong

**Affiliations:** The Center for Vascular Biology, Department of Cell Biology, University of Connecticut Health Center, Farmington, Connecticut, United States of America; Columbia University, United States of America

## Abstract

Here we investigate the role of hypoxia inducible factor (HIF)-2α in coordinating the development of retinal astrocytic and vascular networks. Three Cre mouse lines were used to disrupt floxed *Hif-2α*, including Rosa26^CreERT2^, Tie2^Cre^, and GFAP^Cre^. Global *Hif-2α* disruption by Rosa26^CreERT2^ led to reduced astrocytic and vascular development in neonatal retinas, whereas endothelial disruption by Tie2^Cre^ had no apparent effects. *Hif-2α* deletion in astrocyte progenitors by *GFAP^Cre^* significantly interfered with the development of astrocytic networks, which failed to reach the retinal periphery and were incapable of supporting vascular development. Perplexingly, the abundance of strongly GFAP^+^ mature astrocytes transiently increased at P0 before they began to lag behind the normal controls by P3. Pax2^+^ and PDGFRα^+^ astrocytic progenitors and immature astrocytes were dramatically diminished at all stages examined. Despite decreased number of astrocyte progenitors, their proliferation index or apoptosis was not altered. The above data can be reconciled by proposing that HIF-2α is required for maintaining the supply of astrocyte progenitors by slowing down their differentiation into non-proliferative mature astrocytes. HIF-2α deficiency in astrocyte progenitors may accelerate their differentiation into astrocytes, a change which greatly interferes with the replenishment of astrocyte progenitors due to insufficient time for proliferation. Rapidly declining progenitor supply may lead to premature cessation of astrocyte development. Given that HIF-2α protein undergoes oxygen dependent degradation, an interesting possibility is that retinal blood vessels may regulate astrocyte differentiation through their oxygen delivery function. While our findings support the consensus that retinal astrocytic template guides vascular development, they also raise the possibility that astrocytic and vascular networks may mutually regulate each other's development, mediated at least in part by HIF-2α.

## Introduction

Retinal vascular development is closely associated with the development of an astrocytic template. Earlier studies found that retinal astrocytes were present in animal species with vascularized retinas but absent in animals with avascular retinas [1]. More recent studies employed gene targeting approach in mice to address the relationship between astrocytic and vascular development. *Tlx* null mutation led to poor astrocytic and vascular development in the retina [2]. Besides reduced astrocyte numbers, *Tlx* null mice also displayed poor assembly of extracellular fibronectin matrices [3], and astrocyte specific *Tlx* disruption further demonstrated that the expression of both fibronectin and heparan-sulfate was compromised [4]. These extracellular components were thought to mediate retinal vascularization by regulating VEGF-A binding and distribution [4].

In rodents, the development of retinal astrocyte network begins at birth with immigration of Pax2-positive cells from the optic nerve, spreading in a centrifugal direction in the retinal inner surface [5], [6]. Pax2^+^ cell population gives rise to both optic nerve astrocytes and retinal astrocytes, with the progenitors to the latter also expressing PDGFRα in addition to Pax2 [7]. PDGFRα expression is critical to the proliferation of immature retinal astrocytes in response to stimulation by PDGFA from retinal ganglion cells [8]. As astrocyte progenitors migrate towards the retinal periphery, vascular structures emerge from the optic nerve, forming a vascular network which expands towards retinal periphery behind the PDGFRα^+^ astrocytic network. In vascularized areas, astrocyte maturation occurs, presumably mediated by endothelial cell derived leukemia inhibitory factor (LIF) [9]–[11]. Mature astrocytes exhibit high level expression of glial fibrillary acidic protein (GFAP), whereas Pax2 expression is lost [12]–[14].

How the astrocytic network facilitates retinal vascular development remains incompletely understood. In spite of the important role of VEGF-A in vascular development [15]–[17], astrocyte specific disruption of VEGF-A expression did not interfere with retinal vascular growth, although vascular stability was compromised [18]. Thus, VEGF-A for retinal vascular development is presumably derived from non-astrocytic cells. However, VEGF-A extracellular distribution in developing retinas may be controlled by astrocyte-derived fibronectin and heparan sulfate [3], [4]. In addition, R-cadherin in retinal astrocytes is also important for retinal vascular development, which was demonstrated by blocking R-cadherin function with a neutralizing antibody [19].

A recent study found that HIF-1α deficiency in retinal neural tissues led to compromised development of both astrocytic and vascular networks [20]. In the present study, we compare contributions of HIF-1α and HIF-2α to the development of retinal astrocytic and vascular networks, with an emphasis on the role of HIF-2α in the astrocytic lineage. Selective *Hif-2α* disruption in Pax2^+^ astrocyte progenitor cells led to precocious and accelerated differentiation of Pax2^+^ progenitors into GFAP^+^ astrocytes, causing a shortage in the supply of Pax2^+^ progenitors and premature cessation of astrocyte development. Because HIF-2α protein undergoes oxygen dependent degradation, our findings suggest that retinal vascular development may modulate astrocyte development by regulating Hif-2α protein levels.

## Materials and Methods

### Mice

All animal procedures were approved by the Animal Care Committee at the University of Connecticut Health Center in compliance with Animal Welfare Assurance. Mice were housed with a 12 light/12 darkness cycle, and were maintained on normal chow. Mice were bred by natural mating, and the day when a litter was born was designated P0.

Floxed *Hif-1α* mice were originally produced by Randall Johnson's lab [21] and purchased from the Jackson laboratory in C57BL/6 (B6 from here on) strain background. These mice were crossed with CD1 and subsequently maintained in B6/CD1 mixed background. Floxed *Hif-2α* mice were produced in our own lab from B6/129 hybrid ES cells [22], and then backcrossed to B6 for 4 generations. At this point reproduction slowed down significantly, and a subgroup of the mice were crossed with CD1 females, leading to a population of floxed *Hif-2α* mice in mixed CD1 and B6 background at approximately 50%∶50% ratio. Two *GFAP^Cre^* lines were purchased from the Jackson laboratory, including a line originally generated by Albee Messing (Jax stock number 004600) [23] and another line donated by Michael Sofroniew (line 77.6, Jax stock number 012887) [24]. *GFAP^Cre^* mice originating from the Messing lab were supplied in FVB/N background. These mice were backcrossed into B6 for 4 generations, before they were crossed with CD1 females, resulting in mixed CD1/B6 background similar to that in floxed *Hif-2α* mice. The line 77.6 *GFAP^Cre^* mice from the Sofroniew lab were received in B6 strain background, and were crossed into CD1 by one generation. The tdTomato mice carried a CAG promoter- loxP-Stop-loxP-tdTomato transgene targeted into the ubiquitously expressed *Rosa26* locus [25], and were supplied in B6 strain background. These mice were crossed to CD1 by one generation before being crossed to *GFAP^Cre^* mice. *Tie2^Cre^* was originally generated by Richard Flavell's group [26], and supplied by the Jackson lab (Jax stock number 004128). Detailed breeding information is summarized in Table S1.

Cre recombinases in *Tie2^Cre^* or *GFAP^Cre^* mice were both constitutively active. The Rosa26^CreERT2^ mouse line was a gift from A. Joyner (New York University School of Medicine, New York, New York, USA) and was similar to a related mouse line described by Shebler et. al. [27], [28]. *Rosa26^CreERT2^*-encoded CreERT2 was activated by tamoxifen. Unless specifically indicated, neonatal mice were treated with tamoxifen by daily oral gavage from postnatal day 1 (P1) through P3 (40 mg/kg of body mass, with tamoxifen dissolved in corn oil). In some experiments, pregnant mothers were treated with tamoxifen by oral gavage at 17.5 days post coitum, followed by two more doses delivered to neonates at P1 and P2. In addition, some mice were treated with tamoxifen at P0 through P2. In all cases, pups with or without Rosa26^CreERT2^ were treated with tamoxifen in parallel.

### Genotyping

Mouse ear punch (for weaned mice) and toe clips (for neonatal mice younger than P8) were used for DNA extraction. To determine deletion efficiency in the retina, P5 retinal tissues were used for DNA isolation and PCR analysis. Various alleles were identified using the following primer pairs: *Hif-1α* primers were as described previously [29]; *Hif-2α* primers: EPASloxF (AGTTCTGGCTCCTGCAAGAA) and EPASloxR (TTGCCAGAGGGGAGATGCTAAAATG), wt, 638 bp; floxed, 877 bp; deleted, 260 bp; *Rosa26^CreERT2^* knock in allele was determined as described previously [27]; *Tie2^Cre^* transgene: Tie2CreF (CCGCCTGCTTCTGTGGTG) and Tie2CreR (GCCTGGCGATCCCTGAAC), 260 bp; *GFAP^Cre^* transgene: GFAPCreF (ACTCCTTCATAAAGCCCTCG) and GFAPCreR (ATCACTCGTTGCATCGACCG), 230 bp. All PCR reactions were carried out under the following conditions: 94°C for 9 minutes to activate Taq DNA polymerase (Life Technologies), followed by 35 cycles at 55°C for 1 minute, 72°C for 4 minutes, and 94°C for 30 seconds.

### Whole mount staining of neonatal retinas with isolectin B4 conjugated to Alexa fluor®-594(IB_4_-Alexa 594)

IB_4_ staining of neonatal retinas was carried out as described before [30]. Briefly, neonatal mice were euthanized by decapitation at indicated stages between P0 and P8, and eyes were isolated by enucleation. Isolated eyes were fixed with 4% paraformaldehyde (PFA) for 45 minutes at room temperature, following which retinas were dissected out. Retinas were cut by four incomplete radial incisions, leaving 4 pedals attached to one another at the center. Retinas were incubated at 4°C overnight with IB_4_-Alexa 594 (Life Technologies) at 1∶100 dilution in Retina Staining Buffer (RSB), which consisted of phosphate buffered saline (PBS), 1 mM CaCl_2_, 1 mM MgCl_2_, 1% Triton X100, and 1% BSA. Stained retinas were washed three times in RSB (1 hour each with rocking), and flat-mounted in 50% glycerol in PBS. Imaging was carried out by confocal microscopy.

### Immunofluorescence (IF) staining of flat mount retinas

Retinas were prepared as described for IB_4_-Alexa 594 staining, and incubated with primary antibodies in RSB at 4°C overnight. Primary antibodies included rabbit anti-GFAP (1∶200, Life Technologies), rat anti-GFAP (2 µg/ml, Life Technologies), rabbit anti-Pax2 (1 µg/ml, Life Technologies), and goat anti- PDGFRα (1 µg/ml, R&D Systems). For Pax2 and GFAP double IF staining, rabbit anti-Pax2 and rat anti-GFAP were used. Following incubation with primary antibodies, retinas were washed, and incubated overnight with appropriate secondary antibodies including goat anti-rabbit IgG-Alexa fluor®-488 (1∶200, Life Technologies), donkey anti-rat IgG-Cy3 (2 µg/ml, Jackson ImmunoResearch, , West Grove, PA), and donkey anti-goat IgG-Alexa fluor®-488. Stained retinas were washed thoroughly, and mounted in 50% glycerol in PBS. Images were taken with a Zeiss LSM 510 confocal microscope.

### Detection of proliferative and apoptotic astrocyte progenitors

For proliferation assay, neonatal mice were injected with 5-bromo-2-deoxyuridine (BrdU, 120 mg/kg of body mass; Roche Applied Science, Indianapolis, IN), and euthanized after 60 minutes. Retinas were fixed in 4% paraformaldehyde with the rest of the eyes still attached, and stored in 70% ethanol overnight. Thereafter, fixed retinas were dissected away from other eye structures, treated with 1% Triton X-100 in PBS for 30 minutes at room temperature, and then incubated in 2 mol/L HCl for 1 hour at 37°C. Processed retinas were double stained with mouse anti-BrdU antibody (BD Biosciences, San Jose, CA) and rabbit anti-Pax2, followed by goat anti-mouse IgG-Alexa fluor®-594 and goat anti-rabbit IgG-Alexa fluor®-488.

Apoptosis was monitored by IF staining with anti-active Caspase 3 (Abcam, Cambridge, MA). For positive control, neonatal mice were exposed to 75% oxygen at P7-P8 for 16 hours, returned to room air, and euthanized. Retinas from oxygen exposed mice were dissected, fixed, and stained with anti-active Caspase 3.

### Immunofluorescence staining of retinal cryosections

Retinas prepared as above were embedded in OCT®, and cut at 6 µm. Sections were stained with rabbit anti-GFAP, rabbit anti-Pax2, or mouse anti-neurofilament (anti-NF, University of Iowa Developmental Studies Hybridoma Bank) in RSB for 2 hours. Afterwards, sections were washed, and stained with appropriate secondary antibodies, including goat anti-rabbit IgG-Alexa fluor®-488, or goat anti-mouse IgG-DyLight 488 (1∶200, Jackson ImmunoResearch). After washing and mounting, images were analyzed by confocal microscopy.

### Preparation of nuclear protein extracts and Western blotting

Nuclear protein extract were prepared as described previously [31]. Briefly, neonatal mice were euthanized by decapitation, and retinas were isolated immediately in pre-chilled PBS, which was frequently replaced with additional aliquots of PBS precooled on ice. Nuclear protein extracts were prepared by homogenizing retinas in ice cold nuclear extraction buffer (10 mM HEPES-KOH (pH 7.9), 1.5 mM MgCl_2_, 10 mM KCl, 0.5 mM dithiothreitol, 0.2 mM phenylmethylsulfonyl fluoride, 0.2 mM deferoxamine (Sigma), 0.1% NP-40, and 1× complete protease inhibitor cocktail (Roche)). Nuclei were collected by centrifugation, and resuspended in ice cold re-suspension buffer containing 20 mM HEPES-KOH (pH 7.9), 420 mM NaCl, 1.5 mM MgCl_2_, 0.5 mM dithiothreitol, 0.2 mM deferoxamine, 1× protease inhibitor cocktail, 0.2 mM phenylmethylsulfonyl fluoride, and 25% glycerol. The following antibodies were used for Western blotting: anti-HIF-1α (NB100-449, Novus Biologicals), anti-HIF-2α (NB100-132, Novus Biologicals), and anti-ß-actin (sc-1616; Santa Cruz Biotechnology).

### Statistical analysis

Data were evaluated by two tailed Student's *t*-tests using Excel, and are presented as means ± standard error of means (SEM). All *n* values refer to number of mice. *p*<0.05 was considered significant.

## Results

### Differential but partially overlapping roles of HIF-1α and HIF-2α in retinal vascular development

To compare the roles of HIF-1α and HIF-2α, we generated *Hif-1α^f/f^/Rosa26^CreERT2^* (f = floxed) and *Hif-2α^f/f^/Rosa26^CreERT2^* mice. To activate CreERT2, neonatal mice were treated with tamoxifen by daily oral gavage at P1–P3. This procedure was previously shown to result in highly efficient deletion of floxed exons in a variety of tissues including the neonatal retina [27], [31]. In this study, we were also able to demonstrate significant deletion in retinal tissues (Figure S1).

**Figure 1:**
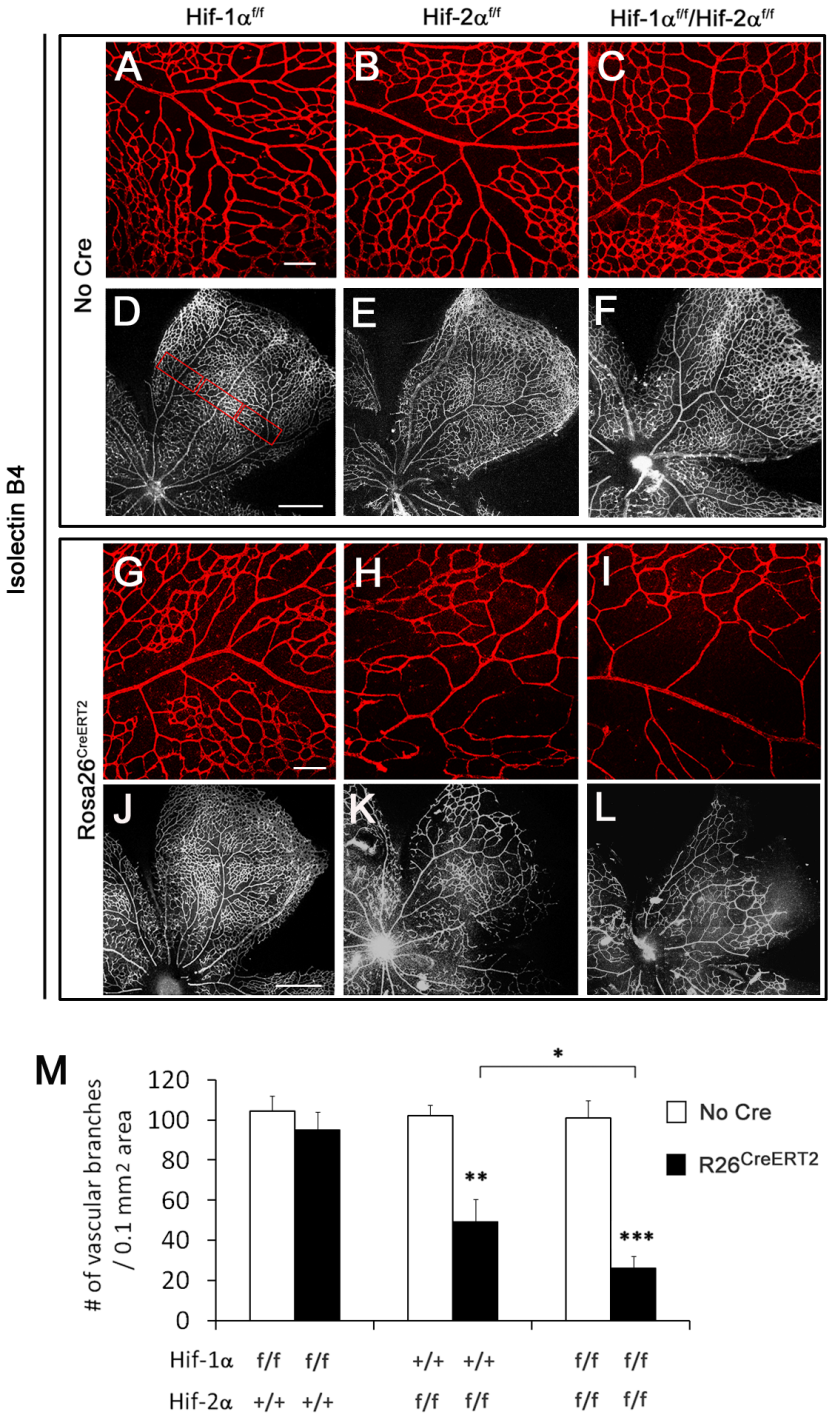
Differential but partially overlapping roles of *HIF-1α* and *HIF-2α* in retinal vascular development. A to L. Confocal images of flat-mounted retinas stained with IB_4_-Alexa 594. A to C and G to I are representative images from areas midway between the optic nerve head and periphery; D to F and J to L each displays one of the 4 pedals of flat-mounted retinas. Images were tiled from multiple panels due to the size of retinas. Genotypes at the *Hif-α* and *Rosa26* loci are indicated on top and to the left of the images, respectively. For example, panels G and J are *Hif-1α^f/f^/Rosa26^CreERT2^*. All mice were treated with tamoxifen by daily oral gavage at P1 through P3 (40 mg/kg in corn oil). At P8, retinas were dissected and stained with IB_4_-Alexa 594. Note that vascular development was reduced in H and K relative to G and J, respective, and further reduced in I and L. Hyaloid vessels were removed during dissection. Scale bars: A and G, 100 µm; D and J, 500 µm. M. Quantification of vascular branches. LoxP modifications at *Hif-1α* and *Hif-2α* loci are indicated below the bar graph, and presence or absence of *Rosa26^CreERT2^* (*R26^CreERT2^*) is indicated by solid or open bars, respectively. Quantification was carried out with the assistance of NIH ImageJ. To average out local variations, vascular branches were counted in 3 rectangular areas (0.2×0.5 mm^2^) areas midway between the optic nerve head and the periphery, with an example shown in D. Where a single pedal was not cut wide enough to encompass all three rectangular areas, the third area was counted in an adjacent pedal. Average values from three areas were used as one data point for statistical analysis. *n* = 6. * *p*<0.05; ** *p*<0.01; *** *p*<0.001.

At P8, the development of primary vascular plexus was examined by IB_4_ staining of retinas from mice treated with tamoxifen at P1–P3. Vascular development was indistinguishable between *Hif-1α^f/f^* and *Hif-1α^f/f^/Rosa26^CreERT2^* mice (Figure 1A, D, G, and J), but was significantly reduced in *Hif-2α^f/f^/Rosa26^CreERT2^* mice (Figure 1B, E, H, and K). The number of vascular branches was quantified in 0.2×0.5 mm^2^ areas midway between the optic nerve head and the periphery (Figure 1M). *Hif-1α^f/f^* and *Hif-1α^f/f^/Rosa26^CreERT2^* mice both had similar number of vascular branches (104.3±7.4 and 95.2±8.7 per 0.1 mm^2^, respectively; *n* = 6, *p* = 0.44). By contrast, *Hif-2α^f/f^/Rosa26^CreERT2^* mice had substantially reduced branching activities (102.1±5.2 in *Hif-2α^f/f^* mice, 49.3±11.1 in *Hif-2α^f/f^/Rosa26^CreERT2^* mice, *n* = 7, *p*<0.01). When tamoxifen treatment was performed at earlier time points (starting at P0 or E17.5), vascular morphogenesis was further reduced in *Hif-2α^f/f^/Rosa26^CreERT2^* mice (Figure S2).

To determine if HIF-1α and HIF-2α might have any overlapping functions, we generated *Hif-1α^f/f^*/*Hif-2α^f/f^/Rosa26^CreERT2^* mice. Vascular development in double disrupted mice was not only defective compared to floxed controls (Figure 1C, F, I, and L), but was also more severely compromised than in *Hif-2α^f/f^/Rosa26^CreERT2^* mice (Figure 1H, I, K and L). While the number of vascular branches in *Hif-2α^f/f^/Rosa26^CreERT2^* mice was about half of floxed controls, the corresponding value in *Hif-1α^f/f^*/*Hif-2α^f/f^/Rosa26^CreERT2^* mice was further reduced to about 26.0% (Figure 1M).

### Apparently normal retinal vascular development following Tie2^Cre^-mediated disruption of floxed Hif-1α or Hif-2α in endothelial cells

Next, we investigated if *Hif-1α* or *Hif-2α* disruption in endothelial cells might cause vascular deficiency. For this test, we used Tie2^Cre^ which was well documented for its high efficiency in deleting floxed DNA sequences in endothelial and hematopoietic cells [26], [32]. For example, we showed in a previous study that Tie2^Cre^ mediated disruption of floxed *Vegfr-1* led to vascular defects identical to those in *Vegfr-1* germline null embryos [30].

**Figure 2:**
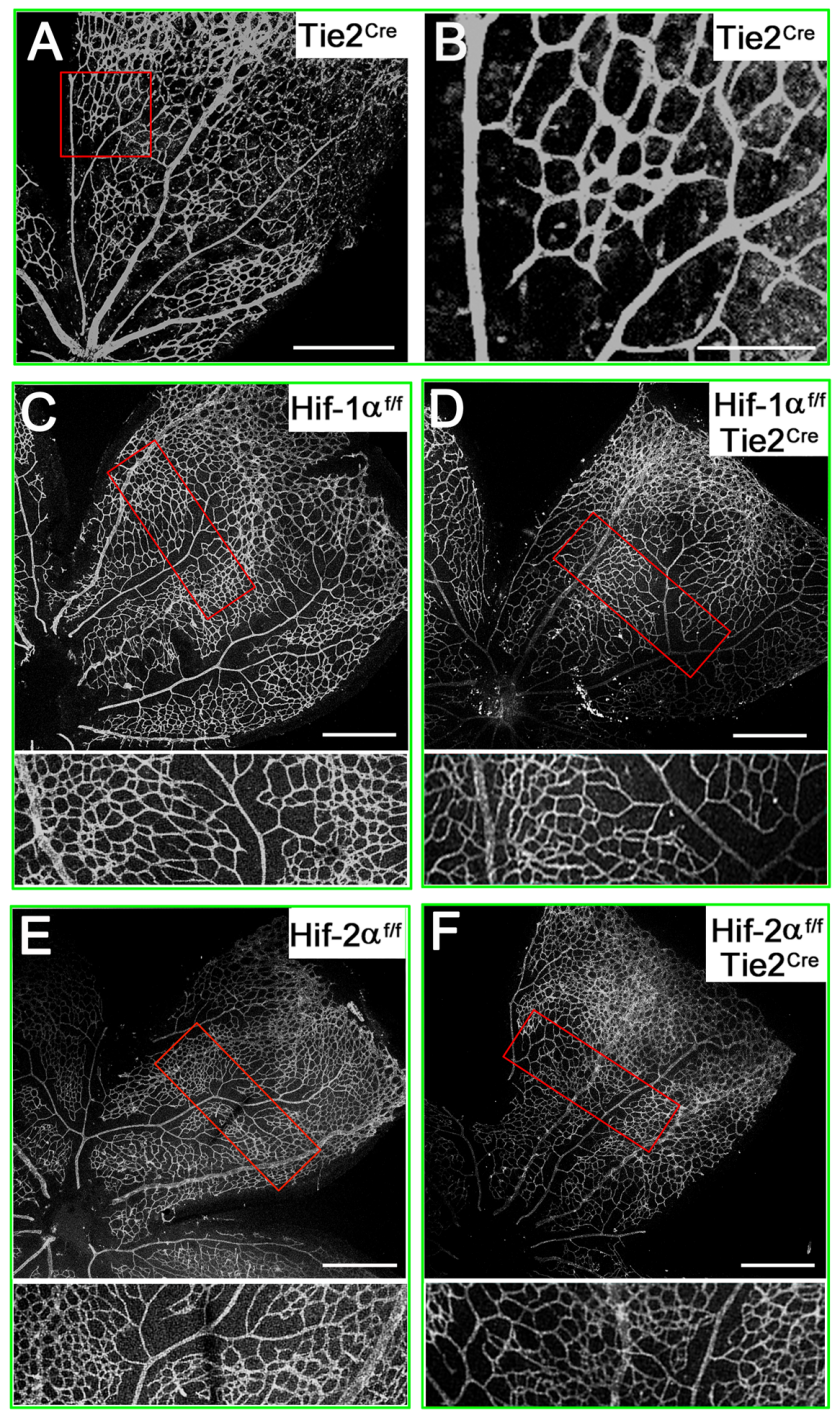
Apparently normal development of the primary layer of retinal vasculature in *Hif-1α^f/f^/Tie2^Cre^* and *Hif-2α^f/f^/Tie2^Cre^* mice. A and B. Confocal images of flat-mounted P7 retinas from tdTomato reporter mice carrying *Tie2^Cre^* transgene. B is expanded from the red rectangle in A. Clearly visible vascular patterns indicate that Tie2^Cre^ is fully functional in retinal blood vessels. C to F. P8 retinas stained with IB_4_-Alexa 594. Areas in red rectangles are shown at higher magnifications below each source image. Images are representative of 4 to 6 mice in each group. Scale bars are 400 µm in A, 125 µm in B, and 500 µm in C–F.

To directly test Cre activity in the context of retinal vascular development, we crossed Tie2^Cre^ mice with tdTomato reporter mice, the latter of which carried a Cre-inducible tdTomato expression cassette integrated into the *Rosa26* locus [25]. Tie2^Cre^ specifically activated the expression of tdTomato in retinal vascular structures (Figure 2A and B), demonstrating that within the retina, Cre activity was most predominantly located in vascular endothelial cells. In *Hif-1α^f/f^/Tie2^Cre^* or *Hif-2α^f/f^/Tie2^Cre^* mice, retinal vascular patterns were indistinguishable from those in floxed mice (Figure 2C to F), suggesting that endothelial HIF-1α or HIF-2α was not essential for normal vascular development in neonatal retinas.

### Reduced development of the astrocytic network in association with HIF-2α deficiency

**Figure 3:**
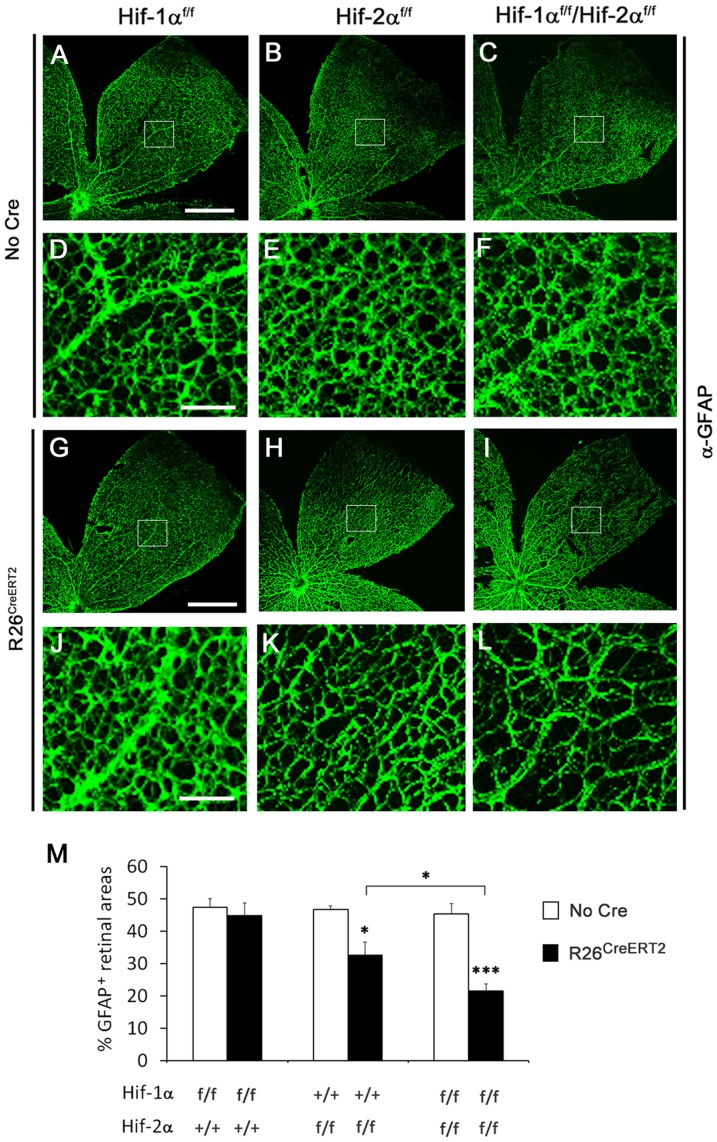
Reduced retinal astrocyte development in *Hif-2α^f/f^/Rosa26^CreERT2^* mice. A to L. Confocal images showing retinal astrocytic networks of P8 neonatal mice. All mice were treated with tamoxifen by daily oral gavage at P1 through P3. At P8, retinas were dissected, fixed, and stained with rabbit anti-GFAP followed by goat anti-rabbit IgG-Alexa 488. Boxed areas in A–C and G–I are shown at higher magnifications below each source image. Scale bars are 500 µm in A and G and 100 µm in D and J. M. Percentage of retinal area occupied by GFAP^+^ cells. Quantification was carried out midway between the optic nerve and periphery (white boxes). Equivalent areas in three different pedals were counted for each retina, and the average value was used as one data point. *Hif-1a* and *Hif-2a* alleles are indicated below each bar; presence of *R26^CreERT2^* is indicated by solid bars. *n* = 6; * *p*<0.05; *** *p*<0.001.

The above findings suggested that the requirement for HIF-α proteins in retinal vascular development might reside in non-endothelial cells. Thus, we examined the development of the retinal astrocytic network by anti-GFAP IF staining in whole mount retinas. At P8, the morphology of astrocytic networks was essentially the same between *Hif-1α^f/f^* and *Hif-1α^f/f^*/*Rosa26^CreERT2^* mice (Figure 3A, D, G, J). However, *Hif-2α^f/f^*/*Rosa26^CreERT2^* mice displayed substantially reduced astrocyte development compared to *Hif-2α^f/f^* mice (Figure 3B, E, H and K). In *Hif-2α^f/f^*/*Rosa26^CreERT2^* mice, retinal tissues were less densely populated with GFAP^+^ astrocytes, and individual astrocyte branches in the network appeared thinner than their counterparts in *Hif-2α^f/f^* mice. Interestingly, astrocyte development was further reduced in *Hif-1α^f/f^*/*Hif-2α^f/f^*/Rosa26*^CreERT2^* mice (Figure 3C, F, I, and L).

The development of astrocytic networks was quantified by calculating the percentage of GFAP^+^ tissues in areas midway between the optic nerve and the periphery (Figure 3M). In *Hif-1α^f/f^* and *Hif-1α^f/f^*/Rosa26*^CreERT2^* mice, GFAP^+^ areas occupied 47.4±2.8% and 44.9±3.8% of total areas, respectively (*n* = 6 per group, *p* = 0.61). In *Hif-2α^f/f^* and *Hif-2α^f/f^/Rosa26^CreERT2^* mice, the corresponding values were 46.7±1.2% and 32.7±3.9% (*n* = 7 per group, *p*<0.05). In *Hif-1α^f/f^*/*Hif-2α^f/f^/Rosa26^CreERT2^* mice, GFAP^+^ area was down to 21.6±2.1%, which was not only significantly lower than 45.4±3.1% in *Hif-1α^f/f^*/*Hif-2α^f/f^* mice (*n* = 7 per group, *p*<0.001), but also lower than in *Hif-2α^f/f^/Rosa26^CreERT2^* mice (*p*<0.05).

### Requirement of astrocyte-derived HIF-2α for vascular development

To determine if HIF-2α was required in astrocytes, we decided to disrupt floxed *Hif-2α* with GFAP promoter-driven Cre. Several different GFAP^Cre^ lines had been reported previously [23], [24], [33], two of which were tested in this study, including line 77.6 from the Sofroniew lab and the line from the Messing lab. To examine Cre activity in neonatal retinas, these Cre lines were crossed with tdTomato reporter mice. Unexpectedly, despite reported expression in astrocytes in the brain, line 77.6 failed to activate any tdTomato expression in the retina (data not shown). In contrast, the line from the Messing lab activated tdTomato expression predominantly in Pax2^+^ astrocyte progenitors and GFAP^+^ astrocytes at P0, P3 and P5 (Figure S3 and Figure S4). Since we have not investigated GFAP^Cre^ activities at developmental stages beyond the focus of this study, our data do not rule out likely GFAP^Cre^ activity in other cell types in older mice.

**Figure 4:**
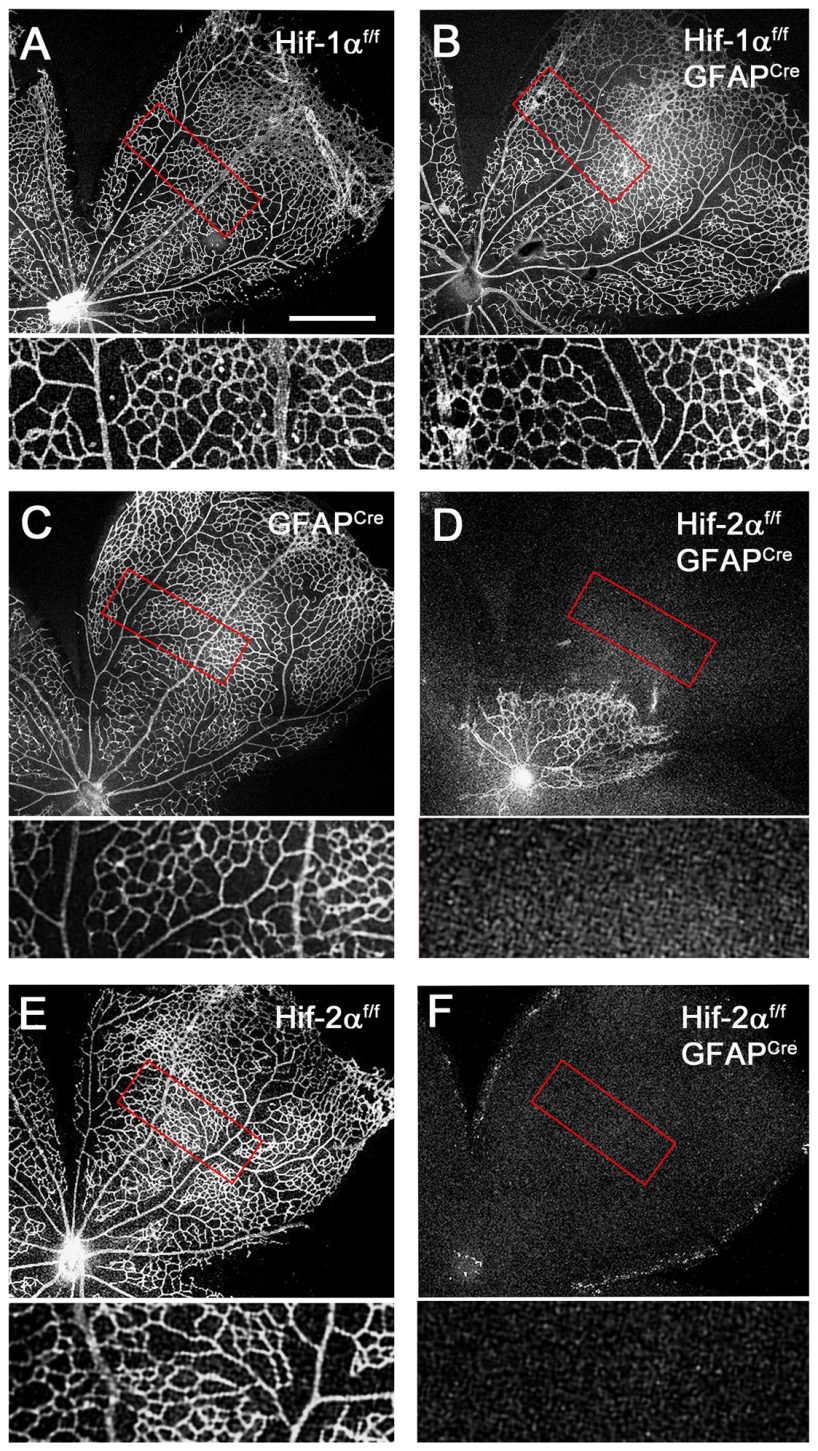
Dramatically reduced retinal vascular development in *Hif-2α^f/f^/GFAP^Cre^* mice. All retinas were dissected at P8, fixed and flat-mounted, and stained with IB_4_-Alexa 594. Development of the primary retinal vascular beds was analyzed by confocal microscopy. A and B. *Hif-1α^f/f^* and *Hif-1α^f/f^/GFAP^Cre^* mice. C to F. *GFAP^Cre^* (C), *Hif-2α^f/f^* (E), and *Hif-2α^f/f^/GFAP^Cre^* (D and F) mice. Areas in red rectangles were expanded and shown below each main image. Out of 22 *Hif-2α^f/f^/GFAP^Cre^* mice analyzed, 2 had remnant amounts of blood vessels, as shown in D, whereas the rest were completely devoid of any vascular development (F). Hyaloid vessels were removed during dissection. Scale bar represents 500 µm, and all main images are in the same magnification scale.

**Figure 5:**
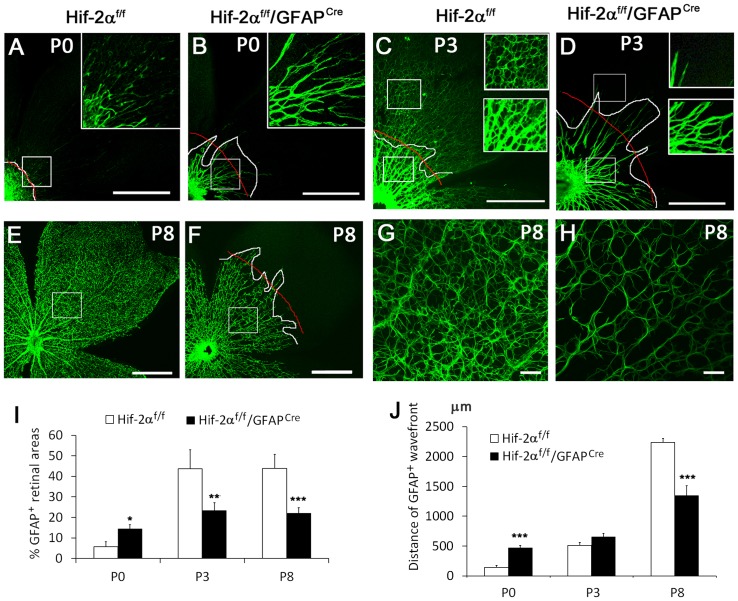
Precocious but tapered development of the astrocytic network in *Hif-2α^f/f^/GFAP^Cre^* mice. Astrocyte development was examined by anti-GFAP IF staining. A and B. Anti-GFAP staining at P0. In A, GFAP^+^ cells are mostly limited to the optic nerve head (lower left). In *Hif-2α^f/f^/GFAP^Cre^* mice, GFAP^+^ structures extended much further. White lines demarcate the borderline between strongly GFAP^+^ and the rest of retinal areas. Red lines mark the approximate average position of the white line. Boxed areas are shown in higher magnifications in the insets. C and D. Anti-GFAP staining at P3. Upper and lower insets are expanded from corresponding boxes. E to H. Anti-GFAP stained retinas at P8 shown at low (E and F) and high (G and H) magnifications. Scale bars, 500 µm in A to F, 50 µm in G and H. I. Percentage (%) of GFAP^+^ areas. Quantifications were carried out in white boxes in A, B, E and F, and lower boxes in C and D. Three such areas were quantified per mouse and average values were used as one data point. J. Quantification of GFAP^+^ wavefronts to the optic nerve, measured as distances between red curves and optic nerves. *n* = 5. * *p*<0.05, ** *p*<0.01, *** *p*<0.001.

We generated *Hif-1α^f/f^/GFAP^Cre^* and *Hif-2α^f/f^/GFAP^Cre^* mice using GFAP^Cre^ mice originated from the Messing lab, and visualized retinal vascular structures at P8 by whole mount staining with IB_4_-Alexa 594. As shown in Figure 4A to C, vascular patterns were similar among *Hif-1α^f/f^*_,_ *Hif-1α^f/f^/GFAP^Cre^* and GFAP^Cre^ mice. In sharp contrast, *Hif-2α^f/f^/GFAP^Cre^* mice displayed drastically reduced vascular development relative to floxed controls (Figure 4D to F). Among 22 *Hif-2α^f/f^/GFAP^Cre^* mice examined at P8, only 2 had remnant amounts of blood vessels as shown in Figure 4D, but the remaining 20 were completely devoid of blood vessels (Figure 5F). While the primary retinal vascular network was absent in most of *Hif-2α^f/f^/GFAP^Cre^* mice, hyaloid vascular structures were abundantly present (Figure S5).

### Precocious but tapered retinal astrocyte differentiation in *Hif-2α^f/f^/GFAP^Cre^* mice

Data presented above strongly pointed to the possibility that retinal astrocyte development might be defective in *Hif-2α^f/f^/GFAP^Cre^* mice. Unexpectedly, retinas from *Hif-2α^f/f^/GFAP^Cre^* mice exhibited significantly more active astrocyte differentiation at P0 (Figure 5A, B, I, and J). Strong GFAP^+^ staining was mostly limited to the optic nerve head in floxed mice but extended much further in *Hif-2α^f/f^/GFAP^Cre^* mice. By P3, the development of astrocytic networks started to lag behind in *Hif-2α^f/f^/GFAP^Cre^* mice (Figure 5C, D, I and J). While strongly GFAP^+^ structures extended similar distances from optic nerves in *Hif-2α^f/f^* and *Hif-2α^f/f^/GFAP^Cre^* mice, astrocytes in the latter failed to form complex network organization and occupied less percentage of retinal areas. Further away from the optic nerve head, weakly GFAP^+^ structures were abundantly present in *Hif-2α^f/f^* but not *Hif-2α^f/f^/GFAP^Cre^* mice (Figure 5C and D, upper rectangles and insets).

By P8, *Hif-2α^f/f^/GFAP^Cre^* mice were completely overtaken by floxed controls (Figure 5E to H). Differences in *Hif-2α^f/f^* and *Hif-2α^f/f^/GFAP^Cre^* mice were manifested in three ways. First, whereas GFAP^+^ cells had spread out over the entire retinal area in *Hif-2α^f/f^* pups, they only reached approximately midway in *Hif-2α^f/f^/GFAP^Cre^* mice (Figure 5E and F). Second, within GFAP^+^ territories, *Hif-2α^f/f^/GFAP^Cre^* mice were less densely populated with astrocytes (Figure 5G, H, I and J). Third, astrocytes in *Hif-2α^f/f^/GFAP^Cre^* mice were much thinner and had fewer branches (Figure 5G and H). In contrast to the dramatic effects of GFAP^Cre^-mediated *Hif-2α* disruption on astrocyte development, little difference was found between *Hif-1α^f/f^* and *Hif-1α^f/f^/GFAP^Cre^* mice. As shown in Figure S6A and B, anti-GFAP staining patterns at P8 were indistinguishable between floxed and *Hif-1α* disrupted mice.

**Figure 6:**
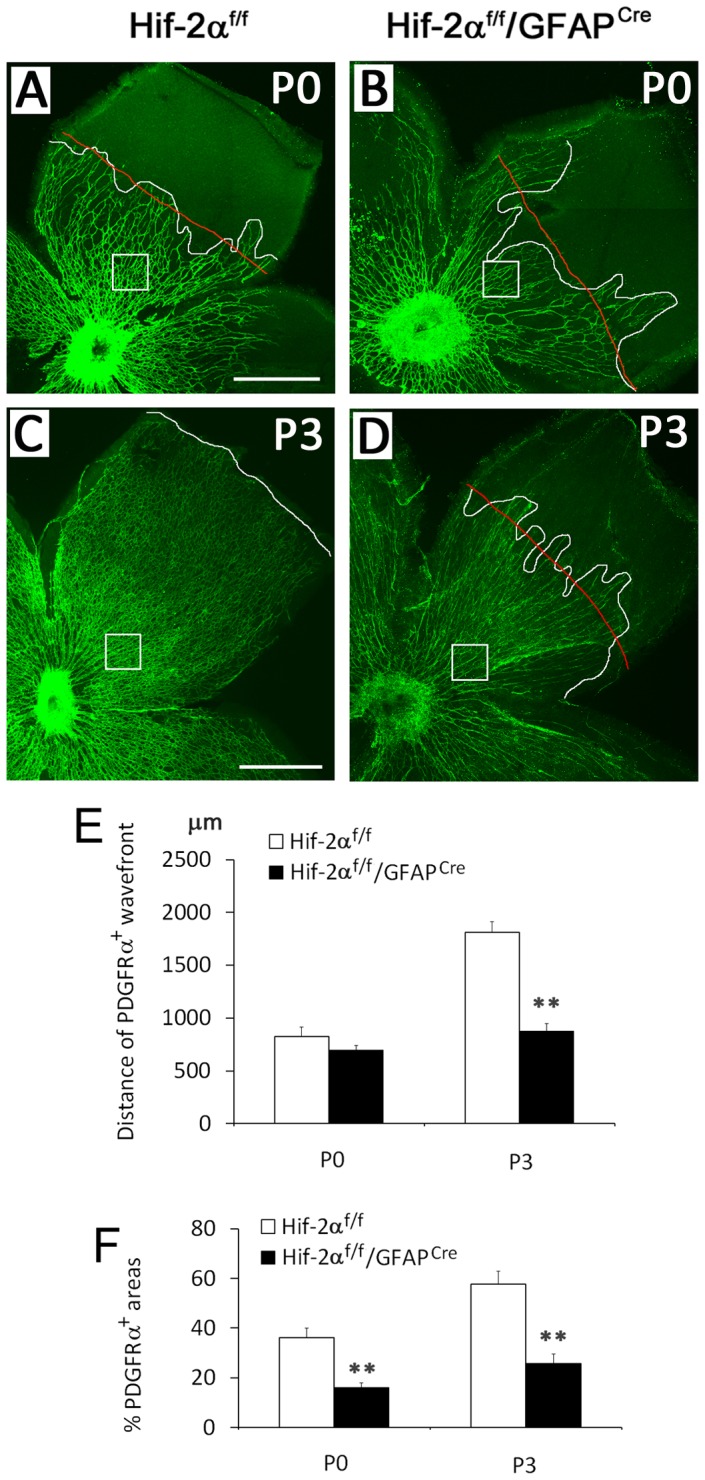
Reduced number of PDGFRα^+^ astrocytes in *Hif-2α^f/f^/GFAP^Cre^* mice. A to D. Retinas dissected at P0 and P3 were subject to anti-PDGFRα IF staining. Wavefronts of PDGFRα^+^ astrocytes are marked by white lines. Red lines represent the approximate average positions of the zigzag white lines. In C, no red line is provided because the front of PDGFRα^+^ astrocyte network was relatively even. Areas indicated by white boxes were quantified for percentage of PDGFRα^+^ tissues. E. Distance from optic nerve head to the front of PDGFRα^+^ areas (white line in C, or red lines in A, B, and D). F. Percentage of PDGFRα^+^ retinal areas. Quantifications were carried for areas indicated by white boxes, taking the average of three such areas from the same mouse as one data point. Scale bars are 500 µm. *n* = 4. ** *p*<0.01.

We also evaluated retinal astrocyte development by anti-PDGFRα staining. At P0, PDGFRα positive astrocyte networks expanded towards the retinal periphery by similar distances in *Hif-2α^f/f^* and *Hif-2α^f/f^/GFAP^Cre^* mice (Figure 6A, B and E). Within PDGFRα^+^ territories however, the percentage of area occupied by PDGFRα^+^ structures is much lower in *Hif-2α^f/f^/GFAP^Cre^* mice (Figure 6A, B and F). At P3, PDGFRα^+^ astrocytes covered essentially the entire retinal inner surface in *Hif-2α^f/f^* mice, but were limited to the more central half in *Hif-2α^f/f^/GFAP^Cre^* mice (Figure 6C, D and E). Within PDGFRα^+^ retinal areas, percentage area occupied by PDGFRα^+^ astrocytes was also significant lower in *Hif-2α^f/f^/GFAP^Cre^* mice (Figure 6C, D and F).

### Depletion of Pax2^+^ astrocyte progenitors by *GFAP^Cre^*-mediated Hif-2α disruption

**Figure 7:**
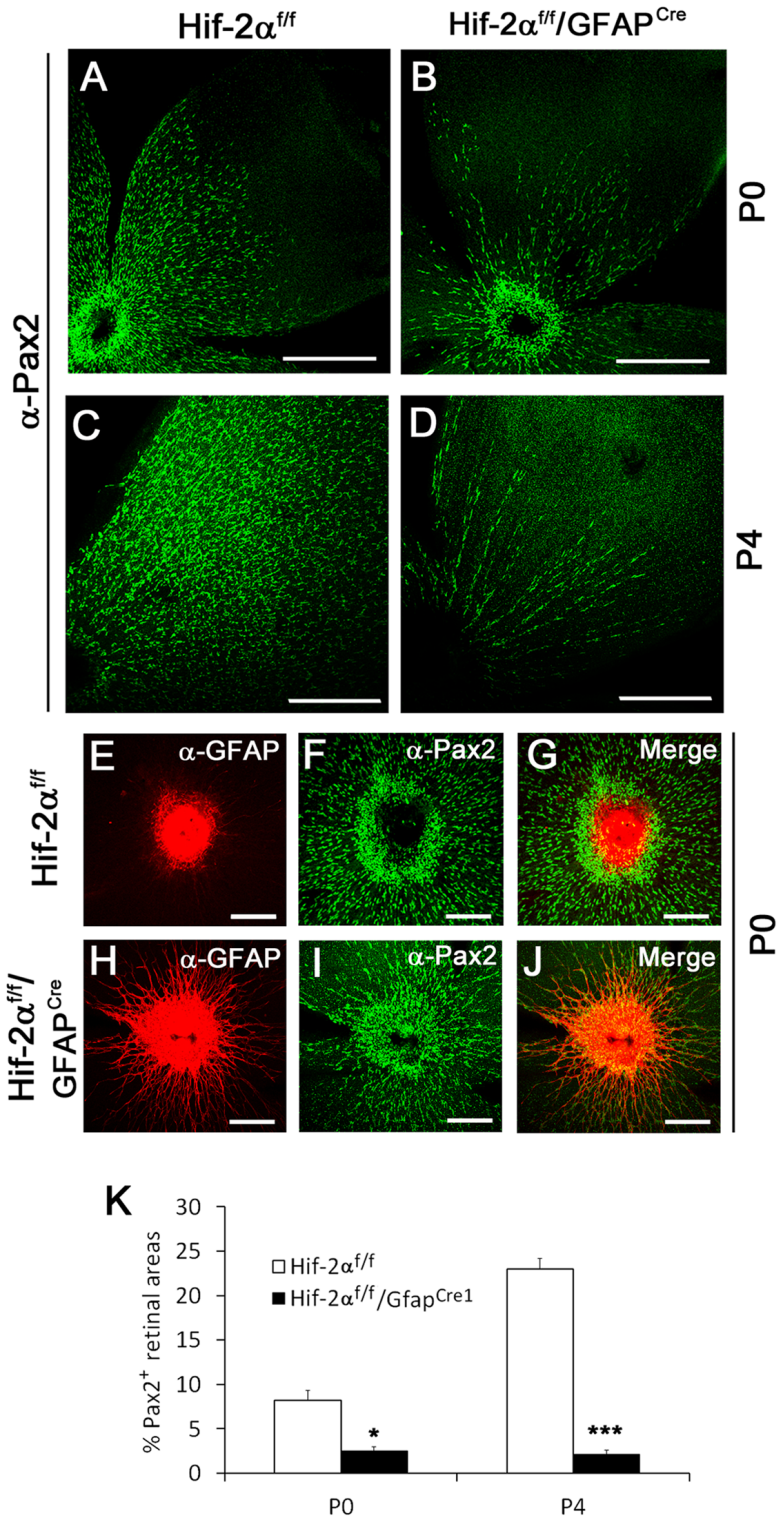
Depletion of Pax2^+^ astrocyte progenitors in *Hif-2α^f/f^/GFAP^Cre^* mice. A to D. Anti-Pax2 IF staining of retinas at P0 (A and B) and P4 (C and D). The abundance of Pax2^+^ cells was dramatically reduced in *Hif-2α^f/f^/GFAP^Cre^* mice at both P0 and P4. Scale bars are 500 µm. E to J. Double IF staining of P0 retinas with rabbit anti-Pax2 and rat anti-GFAP, followed by goat anti-rabbit IgG-Alexa 488 and donkey anti-rat IgG-Cy3. In *Hif-2α^f/f^* retinas, Pax2^+^ (green) are abundantly present in the vicinity of strongly GFAP^+^ (red) optic nerve head, but mature GFAP^+^ astrocytes are virtually absent. In *Hif-2α^f/f^/GFAP^Cre^* mice, Pax2^+^ cells less abundantly present off the optic nerve, accompanied by many GFAP^+^ cells. Scale bars in E to J are 200 µm. K. Percentage (%) of retinal areas occupied by Pax2^+^ cells. *n* = 5. * *p*<0.05, *** *p*<0.001.

To further investigate retinal astrocyte differentiation in *Hif-2α^f/f^/GFAP^Cre^* mice, we analyzed the behavior of Pax2^+^ cells, which represent retinal astrocyte progenitors. At P0, the presence of Pax2^+^ cells was significantly reduced in *Hif-2α^f/f^/GFAP^Cre^* retinas (Figure 7A, B, and K). This difference became more dramatic by P4 (Figure 7C, D and K). We reasoned that increased astrocyte differentiation at early stages might have occurred at the expense of Pax2^+^ progenitor population growth, happening too early and too fast, which resulted in the depletion of astrocyte progenitors and immature astrocytes. To test this hypothesis, we directly compared the development of Pax2^+^ and GFAP^+^ cells by anti-Pax2 and anti-GFAP double IF staining at P0. In *Hif-2α^f/f^* mice, GFAP^+^ signals were mostly limited to the optic nerve head area, but Pax2^+^ cells were abundantly present both near and further beyond the optic nerve head (Figure 7E and F). Pax2^+^ and GFAP^+^ domains did not colocalized, but instead formed a complementary pattern (Figure 7G), demonstrating that Pax2^+^ population was expanding beyond the optic nerve head without significant astrocyte differentiation at this stage of development. In *Hif-2α^f/f^/GFAP^Cre^* littermates, retinas had already developed numerous GFAP^+^ sprouts but were less densely populated with Pax2^+^cells than in *Hif-2α^f/f^* controls (Figure 7H and I). Merged images also revealed that both Pax2^+^ and GFAP^+^ cells were present off the optic nerve head in *Hif-2α^f/f^/GFAP^Cre^* mice (Figure 7J), suggesting that Pax2^+^ cells were quickly differentiating into GFAP^+^ mature astrocytes.

**Figure 8:**
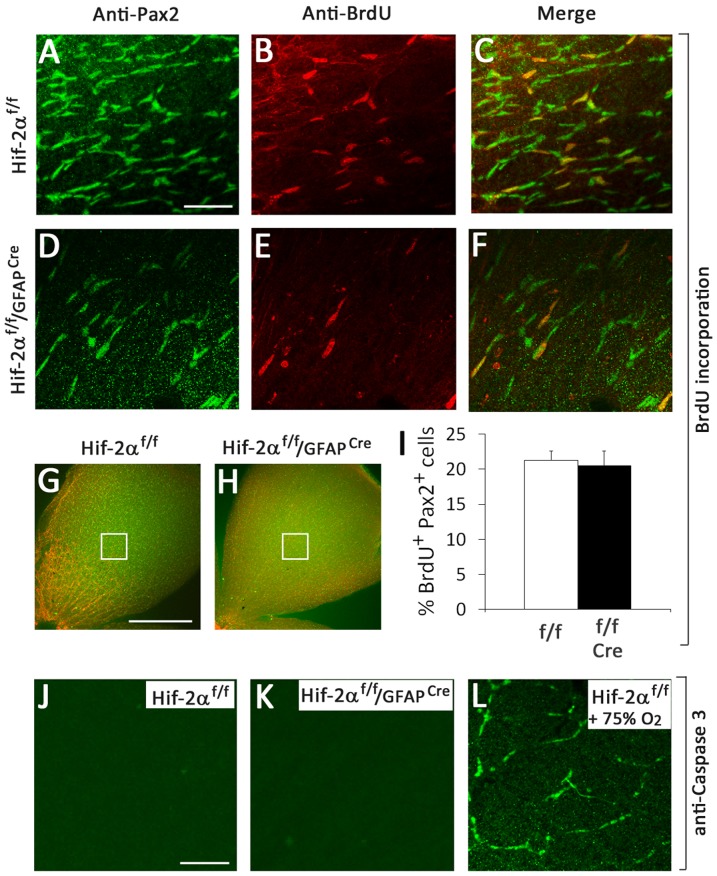
Proliferation and apoptosis assays. A to H. Proliferation assays. Mice were injected with BrdU at P3, and retinas were dissected after an hour. BrdU incorporation in astrocyte progenitors was detected by anti-BrdU and anti-Pax2 double IF staining. A to F show representative images from areas indicated in G and H. Both Pax2^+^ and BrdU^+^ cells were counted with the assistance of NIH ImageJ program. I. Proliferation index was calculated as % of Pax2^+^ cells that were also BrdU^+^. Cre refers to GFAP^Cre^. *n* = 4. No significant difference was found between *Hif-2α^f/f^* and *Hif-2α^f/f^/GFAP^Cre^* mice. J to L. Apoptosis assay by anti-active Caspase 3 IF staining. J and K are P3 retinas. No apoptotic cells were detected in either *Hif-2α^f/f^* or *Hif-2α^f/f^/GFAP^Cre^* mice. L is from mice treated with 75% oxygen for 16 hours between P7 and P8. The same anti-active Caspase 3 staining procedure detected large numbers of apoptotic cells in oxygen-treated mice. Images are representative of data from 4 mice in each group. Scale bars are 50 µm for A to F, 500 µm for G and H, and 50 µm for J to L.

We also assessed proliferation of Pax2^+^ cells at P3. While *Hif-2α^f/f^/GFAP^Cre^* mice contained less abundant Pax2^+^ cells, the percentages of BrdU-labeled Pax2^+^ cells did not differ between *Hif-2α^f/f^* and *Hif-2α^f/f^/GFAP^Cre^* mice (Figure 8A–I). Apoptotic cells were not detectable by anti-active Caspase 3 staining in both *Hif-2α^f/f^* and *Hif-2α^f/f^/GFAP^Cre^* mice (Figure 8J and K). As a positive control, we performed the same analysis in retinas from hyperoxia treated mice. Apoptotic cells were readily detectable by anti-active Caspase 3 staining in the control (Figure 8L). Based on these findings, we conclude that loss of Pax2^+^ cells in *Hif-2α^f/f^/GFAP^Cre^* retinas was unlikely a result of decreased proliferation or increased apoptosis.

## Discussion

### Roles of HIF-α proteins in the development of retinal primary vascular plexus

HIF-1α and HIF-2α have well known roles in angiogenesis in a variety of tissues including embryos, ischemic tissues, and tumors [21], [34]–[41]. In the retina, both HIF-1α and HIF-2α are linked to pathological neoangiogenesis [42]–[44], and HIF-1α in peripheral retinal tissues mediates the development of the intermediate plexus [45]. In this study, global but not endothelial/hematopoietic HIF-2α deficiency led to diminished retinal vascular development. The lack of an essential role for HIF-2α in endothelial cells was not completely unexpected, because mice used in this study were in mixed CD1/B6 background whereas endothelial role of HIF-2α is mostly limited to the 129 strain [35], [36], [46].

HIF-2α deficiency in the astrocytic lineage led to severe vascular defects. This finding contrasts sharply to normal vascular development a previous study which used a different GFAP^Cre^ line to disrupt *Hif-2a* [44]. In the study described here, a GFAP^Cre^ line originally generated in the Messing lab was used [23], which robustly activated the expression of Cre-inducible tdTomato reporter in astrocyte progenitors as early as P0. We presume that early expression of GFAP^Cre^ in astrocyte progenitors may be responsible for the phenotypes presented in this manuscript.

### Regulation of retinal astrocyte development by HIF-2a

In *Hif-2α^f/f^/GFAP^Cre^* mice, astrocytic templates were only partially developed, and the morphology of HIF-2α deficient astrocytes was abnormally thin and elongated. These defects were associated with almost complete absence of retinal vascular development, suggesting that HIF-2α deficiency compromised not only the abundance but also the function of astrocytes. While the co-existence of vascular and astrocytic defects echoes previous findings that the development of retinal vasculature depends on the astrocytic template [2]–[4], a role of HIF-2α in retinal astrocyte differentiation may have further implications on the relationship between retinal astrocytic and vascular development. Since the stability of HIF-2α is sensitively regulated by prolyl hydroxylase domain proteins in an oxygen dependent manner [47]–[50], our findings raise the possibility that regulation of astrocytic and vascular development may be a two way process. In addition to the role of astrocytic templates in regulating retinal vascular growth, their own development may be regulated by oxygen diffusing out of retinal blood vessels. The defects in *Hif-2α^f/f^/GFAP^Cre^* mice may reflect the exaggeration of an inherently important mechanism.

### Interpretation of retinal astrocytic phenotypes in *Hif-2α^f/f^/GFAP^Cre^* mice

Despite reduced astrocyte abundance in *Hif-2α^f/f^/GFAP^Cre^* retinas after P3, astrocyte differentiation from their progenitors was initially increased instead of being decreased, proliferation index of astrocyte progenitors was not reduced, and apoptosis was not increased. These apparently paradoxical phenomena may be understood by proposing precocious and accelerated differentiation of astrocyte progenitors. Because mature astrocytes are non-proliferative, normal development of the astrocytic network depends on the proliferation of astrocyte progenitors and immature astrocytes. If progenitor cells differentiate into mature astrocytes too fast, the supply of the progenitor stock may be depleted because progenitor cells are deprived of the time needed for proliferation. Eventually, dwindling supply of progenitor cells may interrupt astrocyte development. In support of this viewpoint, *Hif-2α^f/f^/GFAP^Cre^* mice indeed contained diminished numbers of Pax2^+^ astrocyte progenitors as soon as mice were born. Since Pax2 and PDGFRα are co-expressed in immature astrocytes [7], it is not surprising that the number of PDGFRα^+^ cells are similar reduced.

The failure of HIF-2α deficient astrocyte network to expand all the way to retinal periphery might also suggest defects in migration. However, this phenotype can be also explained by accelerated differentiation. As retinal development proceeds, the expansion of mature astrocyte network may depend on in situ differentiation from progenitors seeded at progressively more peripheral positions, rather than migration of fully differentiated astrocytes towards the periphery. Indeed, as early as P3, astrocyte progenitors and immature astrocytes have already spread out to essentially the entire inner surface in wild-type retinas (Figure S7). In *Hif-2α^f/f^/GFAP^Cre^* mice, the available astrocyte progenitors were enough to cover only the more central part of the retinas, thus precluding astrocyte differentiation at more peripheral positions.

### The model

**Figure 9:**
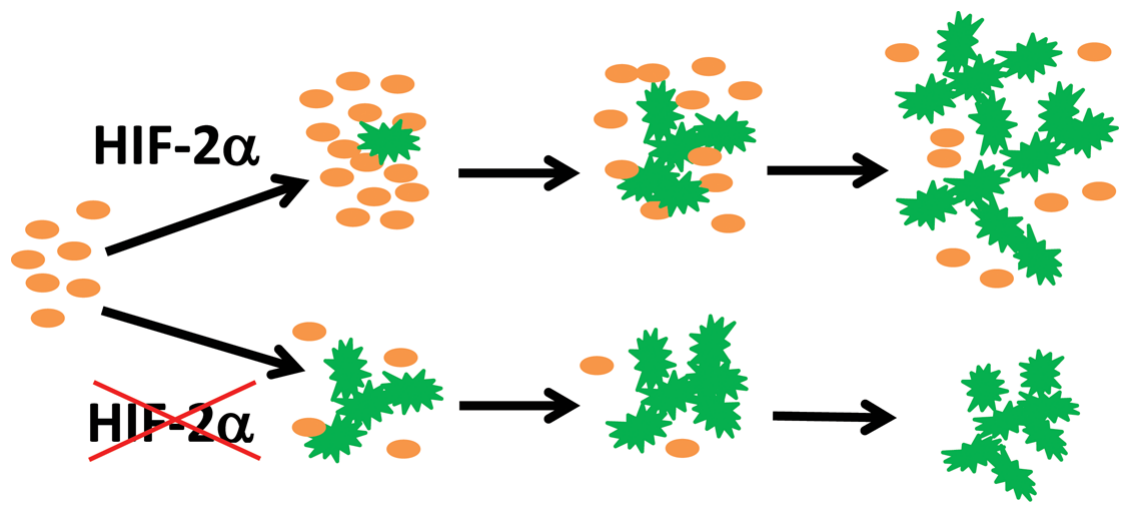
Model for HIF-2α regulated retinal astrocyte differentiation. In wild-type mice, physiological level of HIF-2α may partially suppress differentiation of mature astrocytes (green stars) from their progenitors (orange ovals). Thus, while a proportion of astrocyte progenitors differentiate into non-proliferative mature astrocytes, the rest may undergo active proliferation to replenish progenitor populations. HIF-2α deficiency disturbs the balance between progenitor proliferation and astrocyte differentiation. Accelerated astrocyte differentiation may cause rapid loss of astrocyte progenitors due to inadequate time for proliferation, leading to unsustainable astrocyte development.

Our data led to a model schematically illustrated in Figure 9, which states that HIF-2α regulates retinal astrocyte development by guarding the rate of astrocyte differentiation from immature progenitors. This function is important because astrocyte progenitors are proliferative whereas mature astrocytes are not. Without HIF-2α, accelerated astrocyte differentiation depletes astrocyte progenitors by disallowing them the time needed for proliferation. As a result, retinal astrocyte development ceases prematurely due to the lack of astrocyte progenitors. It should be pointed out that the physiological HIF-2α level in wild-type retinas is apparently insufficient to completely inhibit astrocyte differentiation, but maintains a balance between progenitor population growth and astrocyte maturation.

## Supporting Information

Figure S1 **Disruption of** ***Hif-1α*** **and** ***Hif-2α*** **.** Pups were obtained by crossing *Hif-1α^f/f^/Rosa26^CreERT2^* males with *Hif-1α^f/f^* females, or *Hif-2α^f/f^/Rosa26^CreERT2^* males with *Hif-2α^f/f^* females. At P1–P3, pups were treated with tamoxifen by daily oral gavage. At P5, pups were euthanized, and retinas were dissected. Deletion of floxed *Hif-1α* (A) and *Hif-2α* (B) in retinal tissues was assessed by PCR of retinal DNA extracts. Floxed *Hif-1α*, 260 bp, deleted allele, 270 bp; floxed *Hif-2α* allele, 877 bp, deleted allele, 260 bp. HIF-1α and HIF-2α protein levels were determined by anti-HIF-1α (C) or anti-HIF-2α (D) Western blotting of retinal nuclear protein extracts.
(TIF)

Figure S2 **Induction of more severe vascular defects in** ***Hif-2α^f/f^/Rosa26^CreERT2^*** **mice by tamoxifen treatment at earlier time points.** A and B. *Hif-2α^f/f^* and *Hif-2α^f/f^/Rosa26^CreERT2^* neonatal mice were treated with tamoxifen at P0 through P2. C and D. Pregnant *Hif-2α^f/f^* females mated with *Hif-2α^f/f^/Rosa26^CreERT2^* males were treated with a single dose of tamoxifen at 17.5 d.p.c.. Following birth, neonatal mice were treated with two more doses at P1 and P2. All retinas were stained with IB4 -Alexa 594 at P8. *n* = 3. Scales bar represents 50 µm.
(TIF)

Figure S3 ***GFAP^Cre^*** **activity in retinal astrocytes.** *GFAP^Cre^* mice were crossed with transgenic mice carrying a CAG promoter- loxP-Stop-loxP-tdTomato transgene targeted into the ubiquitously expressed *Rosa26* locus. A to D. Co-localization of tdTomato expression with GFAP^+^ astrocytes. Retinas were dissected from neonatal mice at P5, and cryosections were cut at 6 µm. Sections were stained with rabbit anti-GFAP and anti-Rabbit IgG-Alexa 488, and analyzed by confocal imaging for tdTomato expression (A) or GFAP^+^ cells (B). Merged images are shown in C and D at different magnifications. tdTomato expression and GFAP^+^ cells colocalized at the inner surface of retinal tissues. E to H. Confocal images of tdTomato expression and anti-NF (neurofilament) immunofluorescence staining (green). Cryosections were prepared as in A to D, but stained with mouse anti-NF followed by goat anti-mouse IgG-DyLight 488. It is evident that tdTomato expression does not colocalize with NF^+^ signals. Scales bars, A–C and E to G, 100 µm; D and H, 50 µm.
(TIF)

Figure S4 ***GFAP^Cre^*** **activity in astrocyte progenitors.** *GFAP^Cre^* and Cre-inducible tdTomato transgenic mice were crossed, and pups carrying both transgenes were subject to anti-Pax2 or anti-GFAP IF staining at P0 and P3, and visualized by confocal imaging. The vast majority of tdTomato positive cells were also Pax2^+^ and GFAP^+^, demonstrating early onset of Cre activity in retinal astrocyte progenitors. *n* = 3. All images are at the same magnification. Scale bar represents 50 µm.
(TIF)

Figure S5 **Hyaloid vessels in** ***Hif-2α ^f/f^/GFAP^Cre^*** **mice.** Retinas were dissected at P8, care being taken to preserve hyaloid vessels. A and B. IB_4_-Alexa 594-stained retinas. C and D. Higher magnifications of the top right quarter from A and B, respectively. In C, white arrows point to main arterioles; green arrows indicate large branches between arterioles. Since every main arteriole has just one accompanying venules in normal retinas, additional large branches may be hyaloid vessels. Hyaloid vessels are more prominently present in *Hif-2α ^f/f^/GFAP^Cre^* mice, presumably to compensate for the loss of retinal blood vessels. *n* = 3.
(TIF)

Figure S6 **Apparently normal astrocyte development in** ***Hif-1α^f/f^/GFAP^Cre^*** **mice.** At P8, retinas were dissected from *Hif-1α^f/f^* (A) and *Hif-1α^f/f^/GFAP^Cre^* (B) mice, fixed, and stained by rabbit anti-GFAP followed by goat anti-rabbit IgG-Alexa 488. Data shown are representative confocal images from 4 mice in each genotype. Scales bars are 100 µm. Images are representative of data from 3 mice per group.
(TIF)

Figure S7 **Astrocyte progenitors and immature astrocytes in P3 retinas.** A to C are confocal images of wild-type (*Hif-2α^f/f^*) retinas stained with indicated antibodies at P3. Boxed areas are expanded and shown in D to F. Images in D to F were brightened to show more details at the periphery. These data confirm that by P3, astrocyte progenitors and immature astrocytes are already present throughout the retina. Images are representative of at least 3 mice each. Scales bars are 500 µm for A–C, and 100 µm for D to F.
(TIF)

Table S1 **Breeding strategy and strain background of relevant mouse lines.**
(DOCX)
