# Supplementary figures and images for: Hypoxia Inducible Factor-2α Regulates the Development of Retinal Astrocytic Network by Maintaining Adequate Supply of Astrocyte Progenitors
Source: PLoS One. 2014 Jan 27;9(1):e84736. doi: 10.1371/journal.pone.0084736 (PMC3903483; doi:10.1371/journal.pone.0084736)

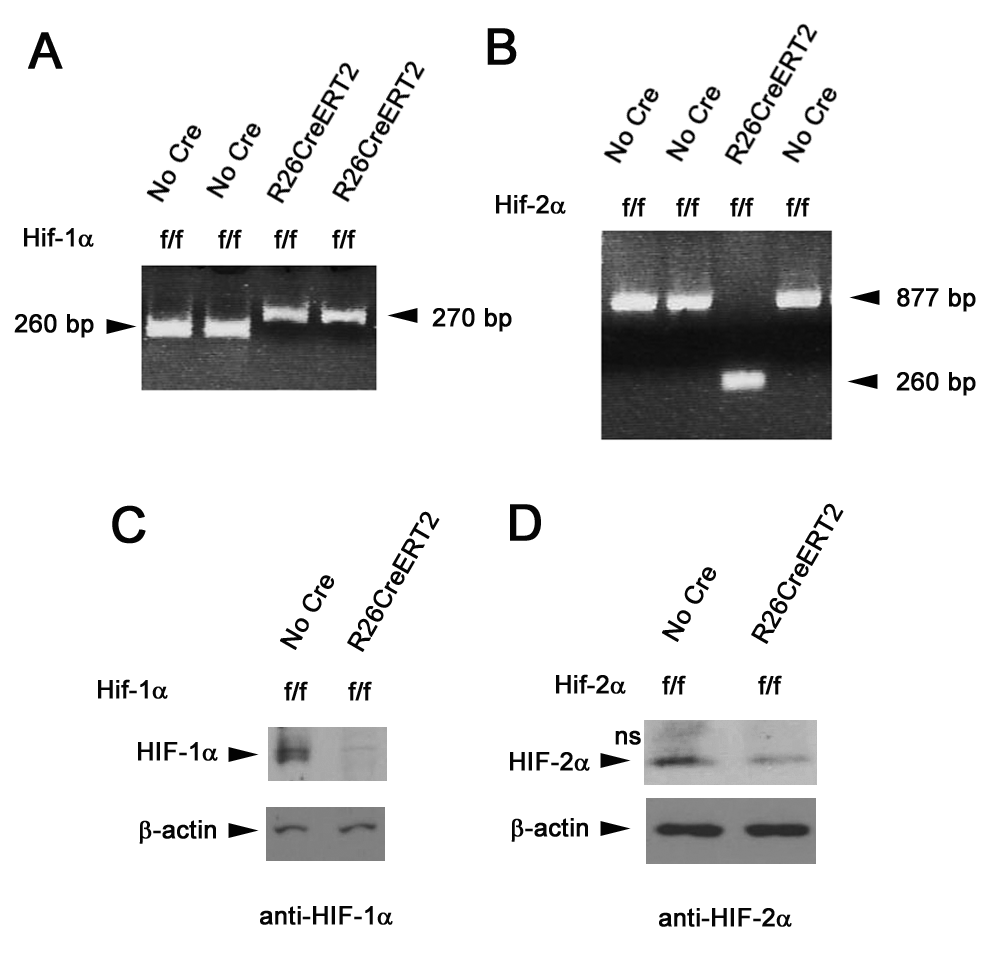

Supplement: Figure S1 — Disruption of Hif-1α and Hif-2α . Pups were obtained by crossing Hif-1αf/f/Rosa26CreERT2 males with Hif-1αf/f females, or Hif-2αf/f/Rosa26CreERT2 males with Hif-2αf/f females. At P1–P3, pups were treated with tamoxifen by daily oral gavage. At P5, pups were euthanized, and retinas were dissected. Deletion of floxed Hif-1α (A) and Hif-2α (B) in retinal tissues was assessed by PCR of retinal DNA extracts. Floxed Hif-1α, 260 bp, deleted allele, 270 bp; floxed Hif-2α allele, 877 bp, deleted allele, 260 bp. HIF-1α and HIF-2α protein levels were determined by anti-HIF-1α (C) or anti-HIF-2α (D) Western blotting of retinal nuclear protein extracts. (TIF) [file pone.0084736.s001.tif]

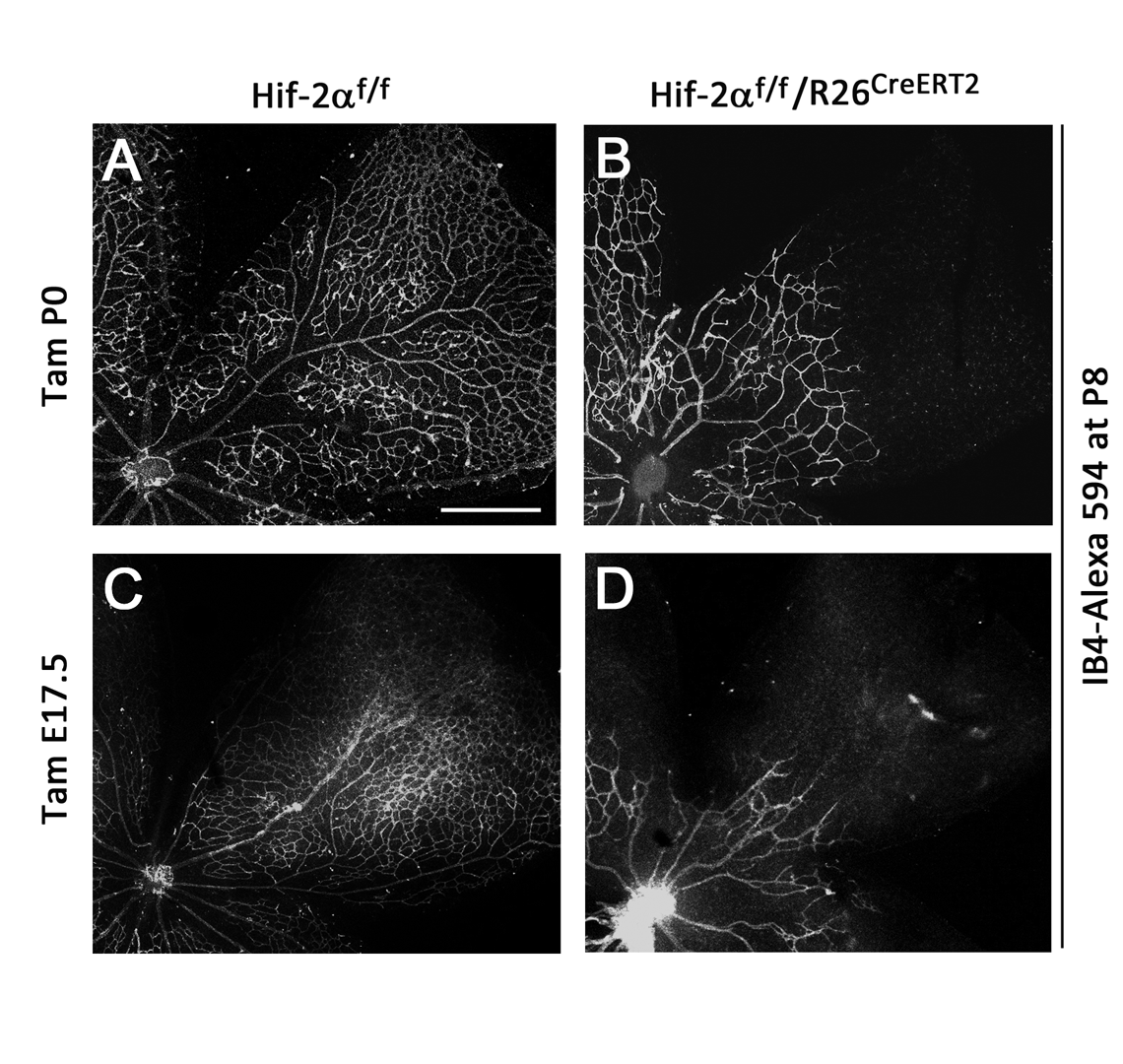

Supplement: Figure S2 — Induction of more severe vascular defects in Hif-2αf/f/Rosa26CreERT2 mice by tamoxifen treatment at earlier time points. A and B. Hif-2αf/f and Hif-2αf/f/Rosa26CreERT2 neonatal mice were treated with tamoxifen at P0 through P2. C and D. Pregnant Hif-2αf/f females mated with Hif-2αf/f/Rosa26CreERT2 males were treated with a single dose of tamoxifen at 17.5 d.p.c.. Following birth, neonatal mice were treated with two more doses at P1 and P2. All retinas were stained with IB4 -Alexa 594 at P8. n = 3. Scales bar represents 50 µm. (TIF) [file pone.0084736.s002.tif]

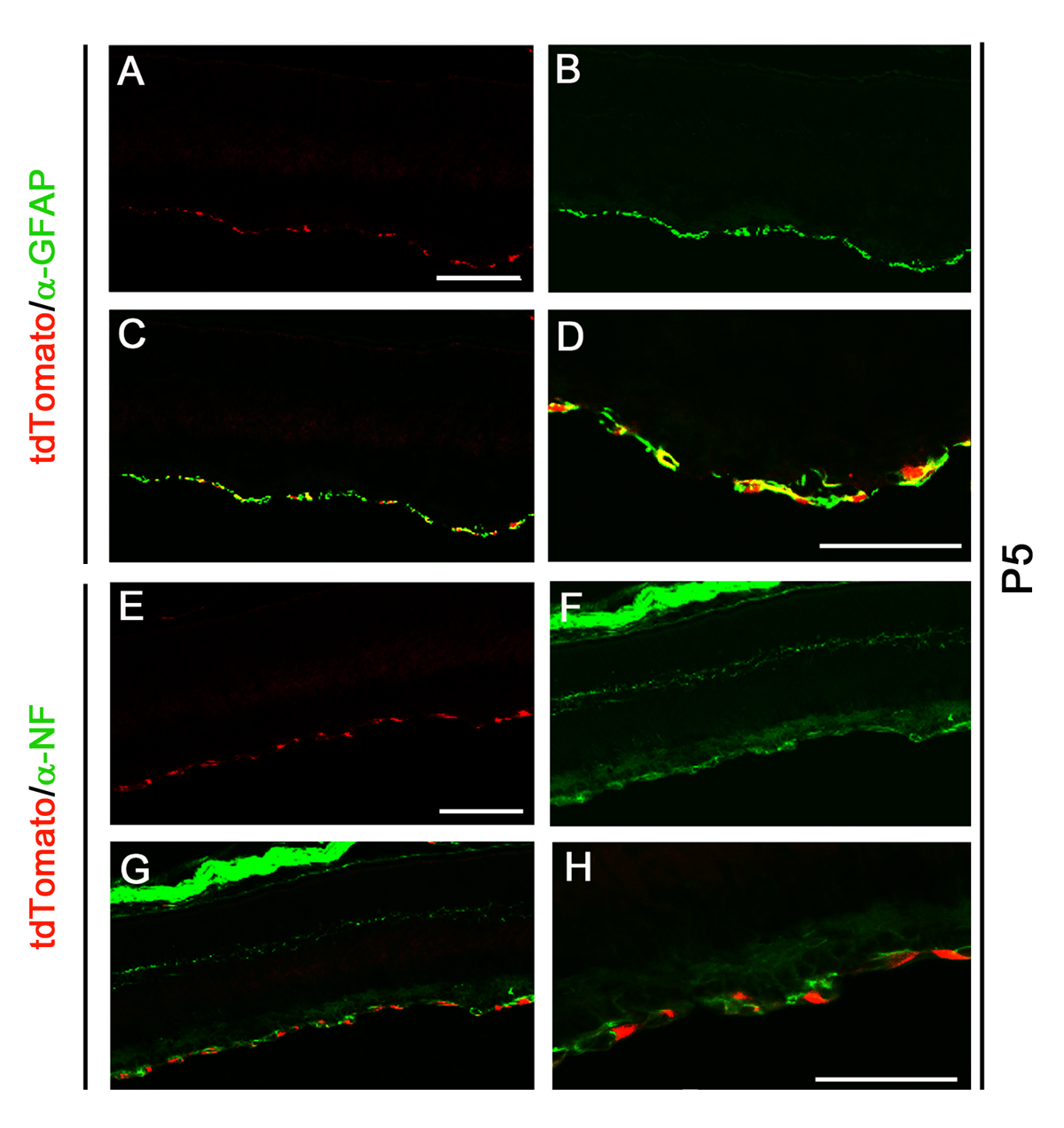

Supplement: Figure S3 — GFAPCre activity in retinal astrocytes. GFAPCre mice were crossed with transgenic mice carrying a CAG promoter- loxP-Stop-loxP-tdTomato transgene targeted into the ubiquitously expressed Rosa26 locus. A to D. Co-localization of tdTomato expression with GFAP+ astrocytes. Retinas were dissected from neonatal mice at P5, and cryosections were cut at 6 µm. Sections were stained with rabbit anti-GFAP and anti-Rabbit IgG-Alexa 488, and analyzed by confocal imaging for tdTomato expression (A) or GFAP+ cells (B). Merged images are shown in C and D at different magnifications. tdTomato expression and GFAP+ cells colocalized at the inner surface of retinal tissues. E to H. Confocal images of tdTomato expression and anti-NF (neurofilament) immunofluorescence staining (green). Cryosections were prepared as in A to D, but stained with mouse anti-NF followed by goat anti-mouse IgG-DyLight 488. It is evident that tdTomato expression does not colocalize with NF+ signals. Scales bars, A–C and E to G, 100 µm; D and H, 50 µm. (TIF) [file pone.0084736.s003.tif]

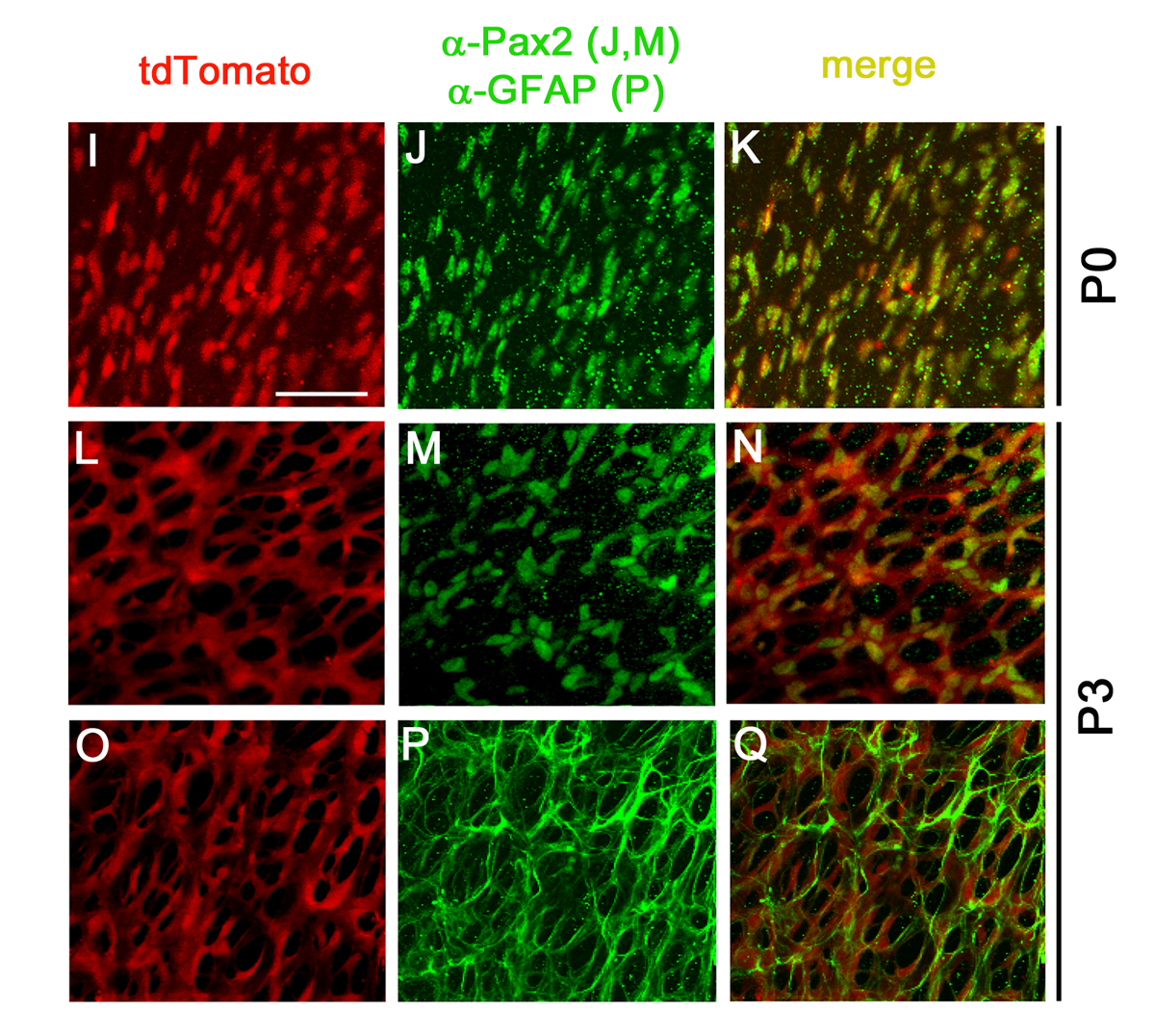

Supplement: Figure S4 — GFAPCre activity in astrocyte progenitors. GFAPCre and Cre-inducible tdTomato transgenic mice were crossed, and pups carrying both transgenes were subject to anti-Pax2 or anti-GFAP IF staining at P0 and P3, and visualized by confocal imaging. The vast majority of tdTomato positive cells were also Pax2+ and GFAP+, demonstrating early onset of Cre activity in retinal astrocyte progenitors. n = 3. All images are at the same magnification. Scale bar represents 50 µm. (TIF) [file pone.0084736.s004.tif]

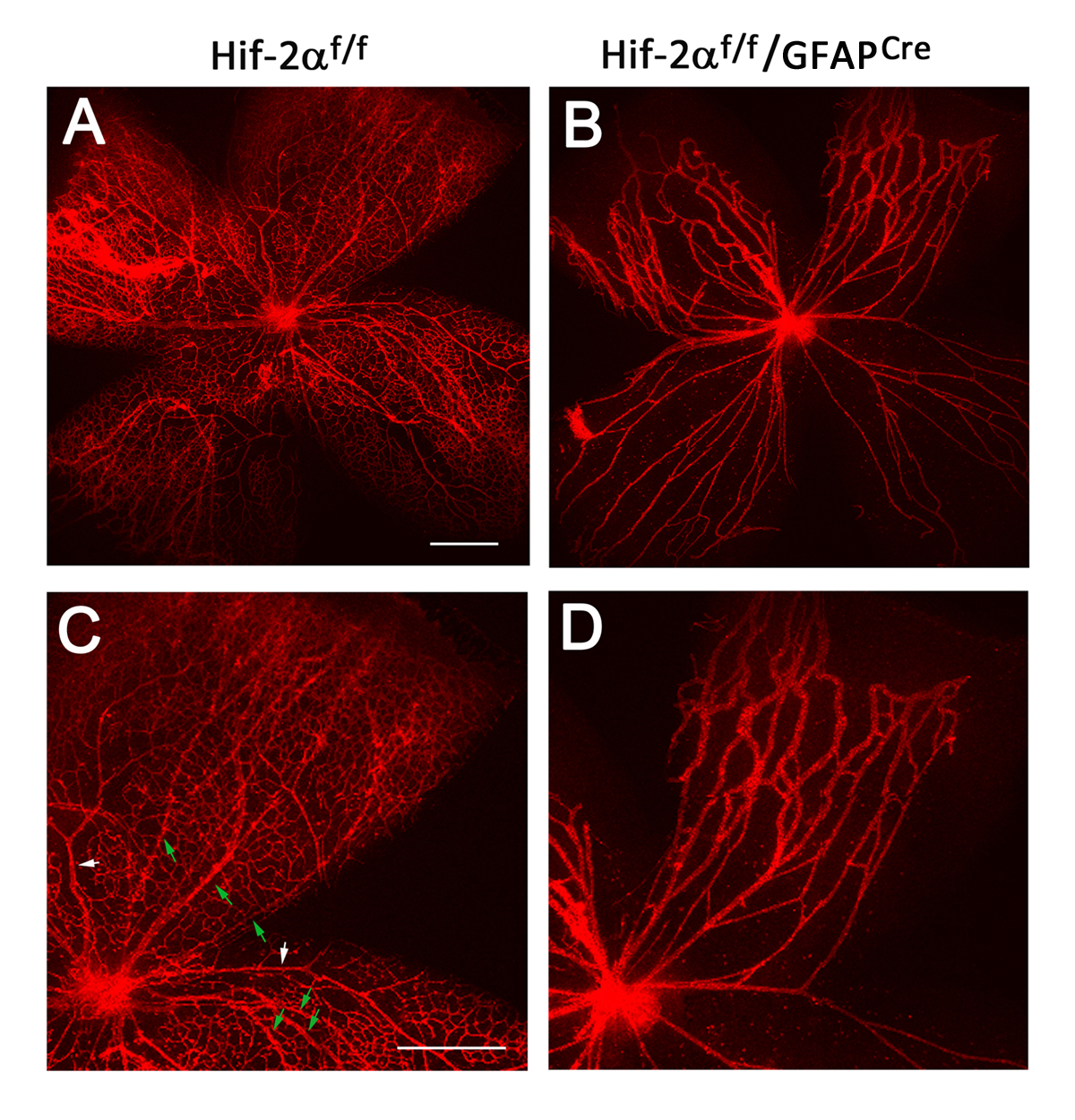

Supplement: Figure S5 — Hyaloid vessels in Hif-2α f/f/GFAPCre mice. Retinas were dissected at P8, care being taken to preserve hyaloid vessels. A and B. IB4-Alexa 594-stained retinas. C and D. Higher magnifications of the top right quarter from A and B, respectively. In C, white arrows point to main arterioles; green arrows indicate large branches between arterioles. Since every main arteriole has just one accompanying venules in normal retinas, additional large branches may be hyaloid vessels. Hyaloid vessels are more prominently present in Hif-2α f/f/GFAPCre mice, presumably to compensate for the loss of retinal blood vessels. n = 3. (TIF) [file pone.0084736.s005.tif]

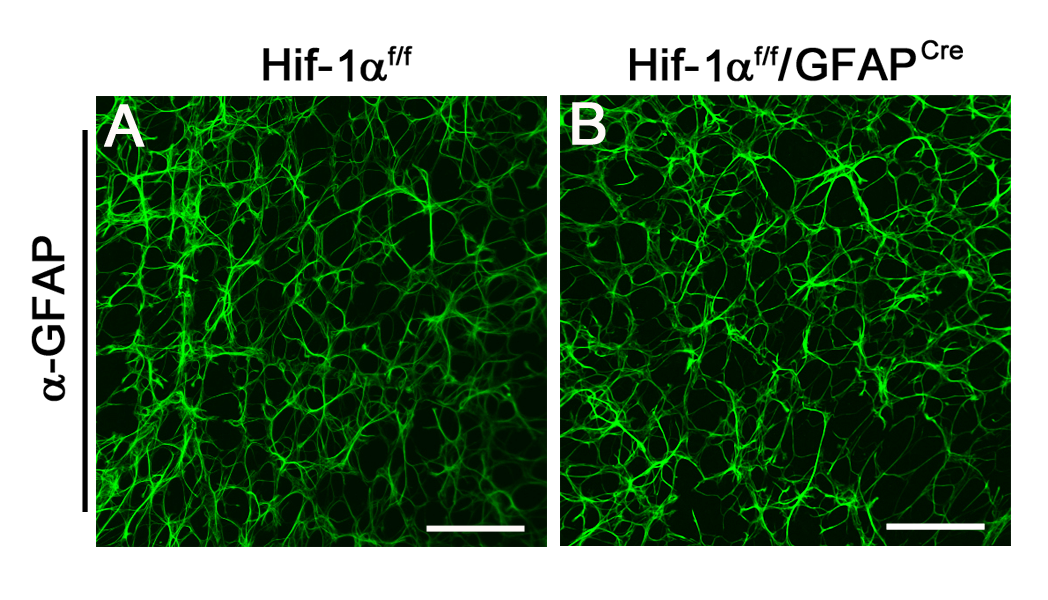

Supplement: Figure S6 — Apparently normal astrocyte development in Hif-1αf/f/GFAPCre mice. At P8, retinas were dissected from Hif-1αf/f (A) and Hif-1αf/f/GFAPCre (B) mice, fixed, and stained by rabbit anti-GFAP followed by goat anti-rabbit IgG-Alexa 488. Data shown are representative confocal images from 4 mice in each genotype. Scales bars are 100 µm. Images are representative of data from 3 mice per group. (TIF) [file pone.0084736.s006.tif]

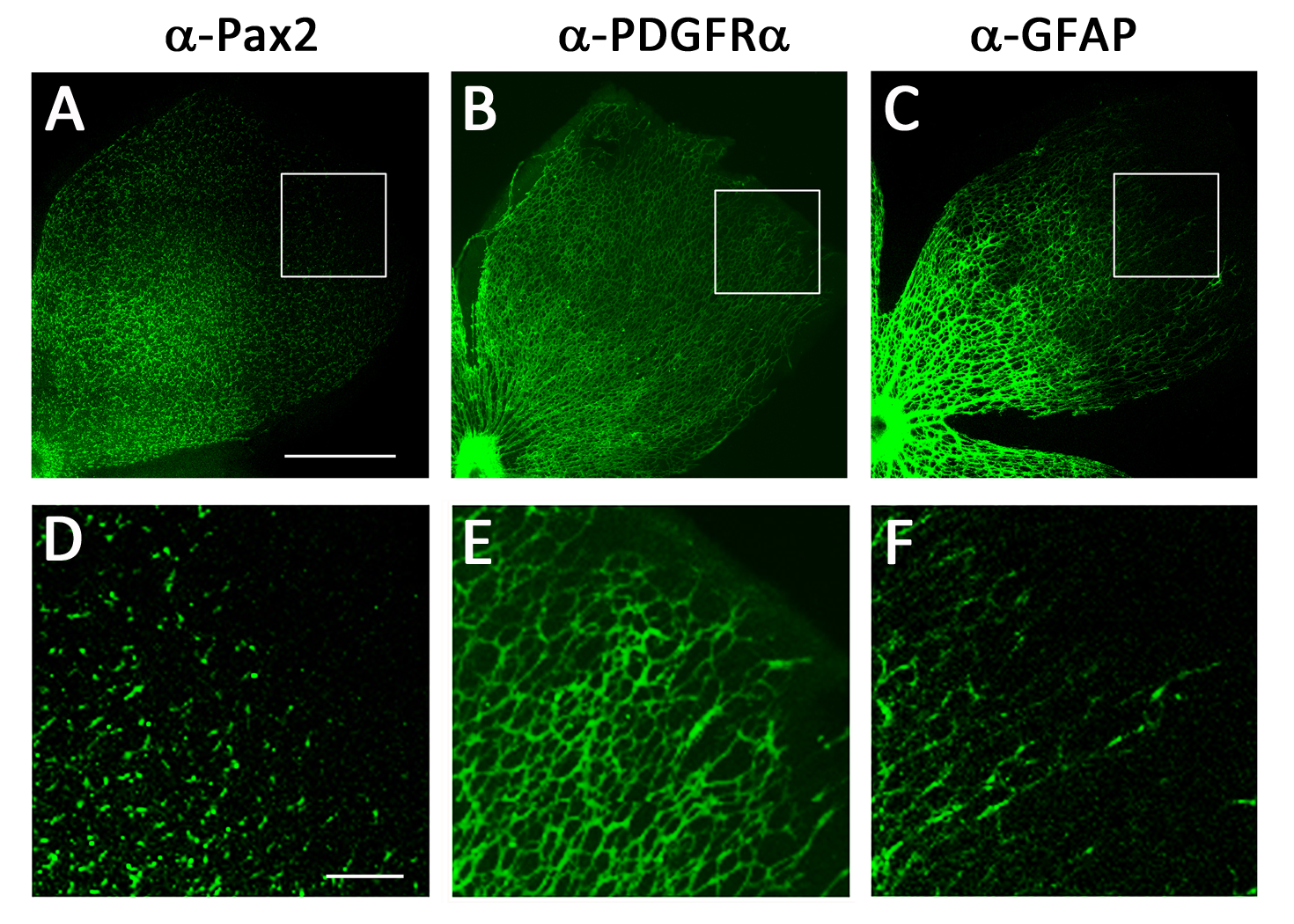

Supplement: Figure S7 — Astrocyte progenitors and immature astrocytes in P3 retinas. A to C are confocal images of wild-type (Hif-2αf/f) retinas stained with indicated antibodies at P3. Boxed areas are expanded and shown in D to F. Images in D to F were brightened to show more details at the periphery. These data confirm that by P3, astrocyte progenitors and immature astrocytes are already present throughout the retina. Images are representative of at least 3 mice each. Scales bars are 500 µm for A–C, and 100 µm for D to F. (TIF) [file pone.0084736.s007.tif]
