# Supplementary material for: Hypoxia Inducible Factor-2α Regulates the Development of Retinal Astrocytic Network by Maintaining Adequate Supply of Astrocyte Progenitors
Source: PLoS One. 2014 Jan 27;9(1):e84736. doi: 10.1371/journal.pone.0084736 (PMC3903483; doi:10.1371/journal.pone.0084736)
Supplement: Table S1 — Breeding strategy and strain background of relevant mouse lines. (DOCX) [file pone.0084736.s008.docx]

| Mouse lines | Originating lab^1^  (references) | Initial Strain background^2^ | Crossing with B6^3^ | Crossing with CD1 | Final strain background |
| --- | --- | --- | --- | --- | --- |
| Floxed Hif-1α | Johnson (22) | B6 | none | 1 generation | CD1/B6 |
| Floxed Hif-2α | Fong (23) | 25% B6,  50% CD1, 25% 129 | 4 generations | 1 generation | CD1/B6 |
| GFAP^Cre^ (line 77.6) | Sofroniew (24) | B6 | none | 1 generation | CD1/B6 |
| GFAP^Cre^ | Messing (23) | FVB | 4 generations | 1 generation | CD1/B6 |
| tdTomato | Zeng (25) | B6 | none | 1 generation | CD1/B6 |
| Tie-2^Cre^ | Flavell (26) | B6 | none | 1 generation | CD1/B6 |

**Table S1. Breeding strategy and strain background of relevant mouse lines.**

^1^ Originating lab refers to the lab where the mouse lines were generated. Most of the above listed mouse lines were obtained from the Jackson Laboratory;

^2^Initial strain background refers to the strain background when mice first arrived at our lab. For floxed *Hif-2α* mice which were generated in our own lab, initial strain background refers to their strain background in the first generation after achieving germline transmission;

^3^ This column refers to the number of generations mice were crossed with B6 *after* they arrived to our lab, or in the case of floxed *Hif-2α* mice, after germline transmission of the floxed allele.
